# A Simple Chinese Risk Score Model for Screening Cardiovascular Autonomic Neuropathy

**DOI:** 10.1371/journal.pone.0089623

**Published:** 2014-03-12

**Authors:** Xiaoli Ge, Shu-Ming Pan, Fangfang Zeng, Zi-Hui Tang, Ying-Wei Wang

**Affiliations:** 1 Department of Anesthesiology, Xin Hua Hospital Affiliated to Shanghai Jiao Tong University School of Medicine, Shanghai, China; 2 Emergence Department, Xin Hua Hospital Affiliated to Shanghai Jiao Tong University School of Medicine, Shanghai, China; 3 Department of Endocrinology and Metabolism, Fudan University Huashan Hospital, Shanghai, China; The Chinese University of Hong Kong, Hong Kong

## Abstract

**Background:**

The purpose of the present study was to develop and evaluate a risk score to predict people at high risk of cardiovascular autonomic dysfunction neuropathy (CAN) in Chinese population.

**Methods and Materials:**

A population-based sample of 2,092 individuals aged 30–80 years, without previously diagnosed CAN, was surveyed between 2011 and 2012. All participants underwent short-term HRV test. The risk score was derived from an exploratory set. The risk score was developed by stepwise backward multiple logistic regression. The coefficients from this model were transformed into components of a CAN score. This score was tested in a validation and entire sample.

**Results:**

The final risk score included age, body mass index, hypertension, resting hear rate, items independently and significantly (P<0.05) associated with the presence of previously undiagnosed CAN. The area under the receiver operating curve was 0.726 (95% CI 0.686–0.766) for exploratory set, 0.784 (95% CI 0.749–0.818) for validation set, and 0.756 (95% CI 0.729–0.782) for entire sample. In validation set, at optimal cutoff score of 5 of 10, the risk score system has the sensitivity, specificity, and percentage that needed subsequent testing were 69, 78, and 30%, respectively.

**Conclusion:**

We developed a CAN risk score system based on a set of variables not requiring laboratory tests. The score system is simple fast, inexpensive, noninvasive, and reliable tool that can be applied to early intervention to delay or prevent the disease in China.

## Introduction

The prevalence of cardiovascular autonomic neuropathy (CAN) is rapidly growing in all populations worldwide, particularly in the developing world. The disease is not only a major factor in the cardiovascular complications of diabetes mellitus (DM) [1], but also affects many other majority segments of general population, such as the elderly, patients with hypertension (HT), metabolic syndrome (MS), and connective tissue disorders [2], [3], [4], [5]. CAN has become a major health concern in China following rapid lifestyle changes. For example, in patients with diabetes, the prevalence of CAN was 30–60% [1]. However, 40% of individuals with CAN were unaware of the disease, as was also found in previous studies [6]. The age at onset of CAN seems to decrease in diabetic or/and hypertension patients. Individuals with previously undiagnosed CAN have an unfavorable cardiovascular risk profile, especially sudden death, indicating a higher risk for cardiovascular disease [7], [8]. Analyses of short- and long-term heart rate variability (HRV) have been proven useful for detecting CA function in patients [9]. CA function testing using HRV is sensitive, noninvasive, and reproducible; therefore, it is easily applicable for screening a large number of individuals in general population [1], [10].

Lifestyle modification has been proven to effectively prevent and delay the development of CAN [1], [6]. Delay and lack of detection of the disease was mostly resulted from patients being asymptomatic during the early stage of the disease so that a simple and accurate screening tool to identify those at high risk of developing CAN will be of great value. It was not be convenient or cost-effective population screening for CAN using 24 hours Holter or Ewing's testing, especially in a resource-poor country. In our previous studies, predictive models for CA dysfunction have been created by using artificial neural network and logistic regression approaches [11], [12]. Furthermore, a simple tool, using a few questions and simple measurement of anthropometric indexes, would be practical for use by the general public and in primary health care. However, a simple CAN risk score based on general Chinese population was little found.

The aim of this study was to develop and evaluate a simple, noninvasive, practical, and informative scoring system to characterize individuals according to their future risk of CAN in Chinese population. A secondary aim was to evaluate the extent of improvement in the score in detecting asymptomatic CAN in a cross-sectional setting.

## Methods and materials

### Study population

This study is a CAN factor survey carried out in a random sample of the middle-aged Chinese population. Participants were recruited from rural and urban communities in Shanghai. The survey participants with undiagnosed CAN, aged 30–80 years, were included in this study. A total of 3,012 subjects were invited to a screening visit between 2011 and 2012. The subjects were excluded from the study to eliminate potential confounding factors that may have influenced cardiac autonomic function [11]. The exclusion criteria were as follows: briefly, 1) history or findings of arrhythmia, hyperthyroidism or hypothyroidism; 2) pregnancy or lactation; 3)serious chronic disease, heart failure and cancer; 4) medication of controlling heart rate such as β receptor inhibitor and 5) seriously liver and renal dysfunction. Of these subjects, complete baseline data were obtained for 2,092 (69.46%) participants without a prior CAN history. Written consent form was obtained from all patients before the study. The present study was approved by the Ethics Committee of the Huashan Hospital, Shanghai, China.

The subjects were interviewed for the documentation of medical histories and medication, history of smoking habits and laboratory assessment of cardiovascular disease risk factors. All study subjects underwent a complete evaluation after 8 hours fast, including: 1) history and physical examination; 2) heart rate, blood pressure; 3) fasting serum glucose and insulin and 4) fasting plasma lipids. Body mass index (BMI) was calculated as the weight in kilograms divided by the square of height in meters. Systolic and diastolic blood pressure (BP) values were the means of two physician-obtained measurements on the left arm of the seated participant. Fasting plasma glucose (FPG) was quantified by the glucose oxidase procedure; HbA1c was measured by ion-exchange high-performance liquid chromatograHTy (HPLC; Bio-Rad, Hercules, CA, USA). Serum total cholesterol (TC), high-density lipoprotein (HDL) cholesterol, triglyceride (TG) levels, creatinine (Cr), and uric acid (UA) were measured by an enzymatic method with a chemical analyzer (Hitachi 7600-020, Tokyo, Japan). Low-density lipoprotein (LDL) cholesterol levels were calculated using the Friedewald formula. The day-to-day and inter-assay coefficients of variation at the central laboratory in our hospital for all analyses were between 1% and 3%.

Short-term HRV has good reproducibility and is more practical for application. In our large-scale population-based study, short-term HRV test was applied to evaluate CA function. Before CA function assessment, participants must avoid alcohol, smoking and coffee for 24 hours so as not to influence their resting status. Subjects were studied while awake, in the supine position after 20 minutes of rest. Testing times were from 8:00 to 11:00 in the morning. A type-I FDP-1 HRV non-invasive detecting system was used with software version 2.0 (Department of Biomedical Engineering of the Fudan University, Shanghai, China). Electrocardiography and respiratory signals and beat-to-beat blood pressure were continually and simultaneously recorded for 15 minutes through an electrosphygmograph transducer (HMX-3C placed on the radial artery of the dominant arm), and an instrument respiration sensor. Short-term HRV analysis was performed for all subjects using a computer-aided examination and evaluation system for spectral analysis to investigate changes in autonomic regulation.

### Definition

HT was defined as blood pressure ≥140/90 mmHg or history of hypertension medication. BMI was classified based on the Chinese criteria: obese [13] BMI≥28.0 kg/m^2^. High fasting plasma glucose (FPG) was defined as FPG≥5.6 mmol/L. High serum triglyceride (TG) was defined as TG≥1.7 mmol/L. Low serum high-density lipoprotein-cholesterol (HDL-C) was defined as HDL-C<0.9 mmol/L in men or HDL-C<1.0 mmol/L in women. Diabetes was defined by oral glucose tolerance test (OGTT) and either HbAlc≥6.5% or the use of insulin or hypoglycaemic medications. MS was diagnosed according to the updated National Cholesterol Education Program/Adult Treatment Panel III criteria (WHO Western Pacific Region obesity criteria) in individuals meeting three or more of the following [14]. CAN was diagnosed based on at least two abnormal cardiovascular autonomic reflex test results [1].

### Statistical analysis

The Kolmogorov-Smirnov test was used to determine whether continuous variables followed a normal distribution. Variables that were not normally distributed were log-transformed to approximate normal distribution for analysis. The results are expressed as the mean ± standard deviation or median, unless otherwise stated. The characteristics of the subjects according to dataset were assessed using one-way analysis of variance for continuous variables and the *χ* ^2^ test for categorical variables.

### Development of the risk score

Potential CAN risk factors known clinically and from the literature to be associated with CAN were selected to be evaluated. The potential risk factors for CAN were age (categorized into three groups: ≤50, 51–60, and >60 years), gender, obesity (BMI≥28.0 kg/m^2^), current smoking (yes/no), resting HR (categorized into three groups: ≤80, 81–90, and >90 beats/min), diabetes, hypertension, blood glucose profile, lipid profile, renal profile. Univariate analyses were performed to estimate significant predictors of CAN. Multiple logistic regression (MLR) was used to compute *β*-coefficients for known risk factors for diabetes. Because the aim was to produce a simple risk calculator that could be conveniently used in primary care and also by individuals themselves, only parameters that are easy to assess without any laboratory tests or other clinical measurements requiring special skills were entered into the model. Variables significant at 5% were included in the MLR using stepwise backward elimination, with CAN as the dependent variable. The independent variables were categorized. P value≤0.05 was considered significant. A scoring system was developed for the simple model; points were assigned to each variable based on the magnitude of its regression coefficient. A sum score was calculated for each participant by adding the score for each variable in the risk model. A receiver-operating characteristic (ROC) curve and area under the curve (AUC) were produced. Sensitivity and specificity were calculated for each cutoff score. The cutoff score that gave the maximum sum of sensitivity and specificity was taken as the optimum [15].

### Validation of the risk score

The performance of the risk score was evaluated in the validation set and entire sample. The predictive performance of the risk score was evaluated using the AUC in ROC curve, sensitivity, specificity, the positive predictive value (the probability of the disease given a positive test), and the negative predictive value (the probability of being non-diseased given a negative test) in the three different sets. The confidence intervals (CIs) for the AUC, sensitivity, specificity, and predictive values were calculated using bootstrapping (1,000) [16]. Furthermore, the proportion of individuals who needed subsequent testing (the proportion of individuals who have a score above the selected cutoff value in the risk score) was compared.

## Results

The baseline clinical characteristics of the 2092 subjects were listed in Table 1. The entire sample included 905 male and 1187 women (mean age, 60.78±9.25 years), and 18.51% of these individuals were found to have CAN. There were more than 80% of the total individuals aged 50–70 years old. The mean BMI was 24.21 kg/m^2^. The mean FPG, TC and TG were 5.53, 5.32 and 1.71 mmol/L, respectively. HRV components were decreased with aging. The majority of subjects had never smoked (85.37%), and the prevalence of HT, DM and MS were 46.65%, 21.33% and 39.82% in the entire sample, respectively. The CAN prevalence was 31.17%, 24.69%, 24.49% and 24.14% in patients with diabetes, hypertension patients, MS patients and individuals aged ≥60 years old, respectively. 1054 individuals and 1038 individuals were randomly selected to be exploratory set and validation set. The prevalence of CAN was 17.65% and 19.36% in exploratory set and validation set, respectively. The baseline characteristics were similar between exploratory and validation set (p<0.05, Table 1).

**Table 1:** Characteristics of subjects.

	Exploratory Set	Validation Set	Entire sample	*P* value*
N	1054	1038	2092	-
Demographic information				
Age	60.65±8.61	60.18±8.76	60.42±8.68	0.206
Gender male,%	440(41.75%)	465(44.80%)	905(43.26%)	0.397
BMI Kg/m^2^	24.10±3.32	24.35±3.43	24.21±3.37	0.154
SBP mmHg	127.48±18.8	127.76±18.76	127.62±18.77	0.737
DBP mmHg	79.89±10	79.77±9.47	79.83±9.74	0.777
Laboratory assays				
FPG mmol/L	5.55±1.80	5.51±1.83	5.53±1.82	0.457
PBG mmol/L	7.64±3.60	7.71±3.67	7.67±3.63	0.793
TC mmol/L	5.32±1.01	5.32±1.01	5.32±1.00	0.766
TG mmol/L	1.67±0.99	1.75±0.97	1.71±0.98	0.214
HDL mmol/L	1.35±0.33	1.36±0.32	1.36±0.32	0.248
LDL mmol/L	3.17±0.77	3.19±0.77	3.19±0.77	0.934
UA µmolL	279.12±84.01	283.38±83.99	281.21±84.01	0.249
HRV Components				
HR beats/min	72.13±9.96	72.71±10.3	72.42±10.13	0.224
TP ms^2^	882.00±706.78	865.59±698.21	873.95±702.47	0.595
LF ms^2^	188.22±193.57	193.84±221.82	190.98±207.88	0.538
HF ms^2^	185.53±216.37	180.48±222.65	183.05±219.43	0.599
LF/HF	1.67±1.91	1.72±2.06	1.7±1.98	0.647
Medical history				
Smoking yes,%	140(13.28%)	166(15.99%)	306(14.63%)	0.111
CAN yes,%	186(17.65%)	201(19.36%)	387(18.51%)	0.366
MS yes,%	417(39.56%)	416(40.08%)	833(39.82%)	0.698
HT yes,%	500(47.44%)	476(45.86%)	976(46.65%)	0.621
DM yes,%	231(21.92%)	215(20.71%)	446(21.33%)	0.467

Note:

* present the difference between exploratory set and validation set.

BMI- Body mass index, WC-waist circumference, SBP- systolic blood pressure, DBP- diastolic blood pressure, FPG- fasting plasma glucose, PBG- plasma blood glucose, TC- serum total cholesterol, TG- triglyceride, UA- uric acid, HDL- high-density lipoprotein cholesterol, LDL- low density lipoprotein cholesterol, Ccr- creatinine clearance rate, HR-heart rate, TP-total power of variance, LF-low frequency, HF-high frequency, MS- metabolic syndrome, HT- Hypertension, DM- Diabetes.

### Model development

**Figure 1:**
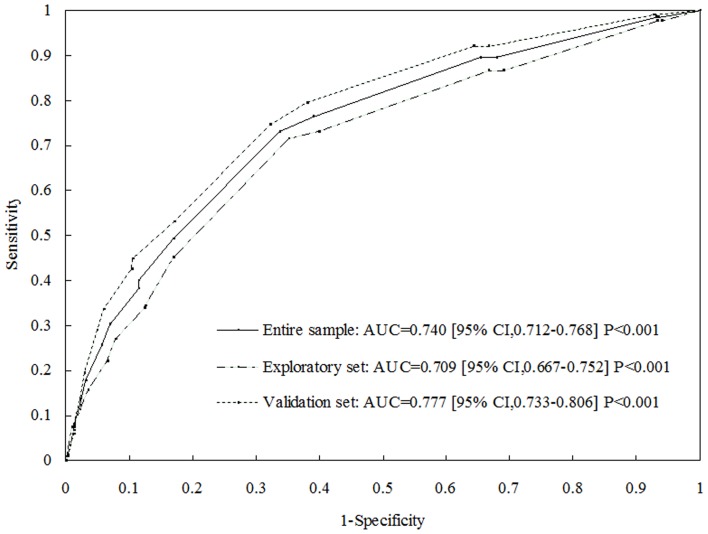
Receiver operating characteristic curves showed the performance of each cardiovascular autonomic neuropathy (CAN) risk score (CRS) in predicting prevalence of diabetes in the exploratory, validation and entire cohorts. The 95% confidence interval (CI) is given in parentheses. AUC - area under the curve.

Univariate association analysis to include potential risk factors showed that resting HR, DM, SBP, DBP, HT, BMI, Age, and TG were significantly associated with CAN (P<0.05 for all, Table 2). In the simple multivariable regression model, using all these variables except DM, FPG and PBG, after stepwise backward elimination of the non-significant variables, the final multivariable regression model included risk factors of age, HT, and HR (P<0.05 for all Table 3). Plausible interactions were tested between age and BMI, age and HT, age and HR, BMI and HT, BMI and HR, and HT and HR, but none of these were significant (data not shown). HR was the strongest predictor for CAN followed by HT and BMI. The score for each variable is shown in Table 3. The total score ranged between 0 and 15. Cutoff value of the sum score 5 showed a sensitivity value with a little greater than 70% but at a little higher proportion (44.88%) that needed subsequent standard HRV tests (Table 4). Both the sensitivity and the proportion that needed subsequent standard HRV testing were decreased with increasing the cutoff value. Because a sensitivity of 70% was expected, no more than 40% of the entire population might need subsequent testing. The cutoff score of 6 was optimum (sensitivity = 70.97%; specificity = 67.40%; Youden index = 38.37% and % need subsequent testing = 39.88%, Table 4). The AUC was 0.709 (95% CI 0.667–0.752, Figure 1), which is almost identical to that for the corresponding model. The cutoff value of 6 out of 15 points was chosen for evaluation.

**Table 2:** Univariate analysis of risk factors for CAN (ranking by coefficient).

Variable	β	S.E.	*P* value	OR (95%CI)
HR	0.983	0.126	<0.001	2.67 (2.09–3.42)
DM	0.816	0.175	<0.001	2.26 (1.61–3.19)
HT	0.727	0.116	<0.001	2.07 (1.49–2.86)
PBG	0.706	0.165	<0.001	2.03 (1.47–2.80)
DM duration	0.623	0.127	<0.001	1.86 (1.45–2.39)
MS	0.592	0.161	<0.001	1.81 (1.32–2.48)
Age	0.563	0.136	<0.001	1.76 (1.35–2.29)
FPG	0.535	0.167	0.001	1.71 (1.23–2.37)
BMI	0.404	0.065	<0.001	1.50 (1.32–1.70)
TG	0.300	0.163	0.046	1.35 (0.98–1.86)
HT duration	0.289	0.068	<0.001	1.34 (1.17–1.53)

Note: HR-heart rate, FPG- fasting plasma glucose, PBG- plasma blood glucose, TG- triglyceride, BMI-body mass index, HT- Hypertension, DM- Diabetes, MS-metabolic syndrome.

**Table 3:** CAN risk score based on the simple model in the exploratory set.

Variable	*β*	*P* value	OR (95%CI)	Risk score*
Age (year)				
≤50	0.000	-	1.00	0
50–60	0.438	0.002	1.55 (1.18–2.04)	2
>60	0.876	<0.001	2.40 (1.82–3.17)	4
BMI (kg/m^2^)				
<28.0 kg/m^2^	0.000	-	1.00	0
≥28.0 kg/m^2^	0.236	0.043	1.27 (1.01–1.58)	1
Hypertension				
No	0.000	-	1.00	0
Yes	0.574	0.001	1.78 (1.26–2.51)	2
Heart Rate (beats/min)				
≤80	0.000	-	1.00	0
81–90	0.957	<0.001	2.60 (2.02–3.35)	4
>90	1.914	<0.001	6.78 (5.27–8.73)	8

Note:

*For each significant variable in the multiple logistic regression analysis, a risk score was calculated from the regression coefficients (*β*) dividing by a common factor (0.236) and rounding to the nearest integer.

**Table 4:** Performance of the risk score in the three different cohorts.

Data Set	Sensitivity (%)	Specificity (%)	Youden Index (%)	PPV (%)	NPV (%)	%need testing*
Exploratory Set						
cutoff = 5	73.12(70.47–75.77)	61.18(54.73–67.63)	34.30(30.28–38.33)	29.95(28.05–31.85)	90.93(90.43–91.43)	44.88(43.28–46.48)
cutoff = 6	70.97(68.21–73.73)	67.40(60.48–74.32)	38.37(34.01–42.73)	33.07(30.73–35.41)	91.09(90.14–92.05)	39.73(38.09–41.37)
cutoff = 7	39.78(36.7–42.86)	85.37(78.7–92.04)	25.15(20.75–29.55)	38.16(36.19–40.14)	86.2(85.48–86.91)	19.07(17.16–20.98)
Validation Set						
cutoff = 5	77.61(74.78–80.44)	63.92(57.39–70.45)	41.53(37.4–45.66)	31.67(29.55–33.8)	92.98(92.28–93.68)	44.12(42.04–46.2)
cutoff = 6	74.63(71.6–77.66)	67.50(60.84–74.16)	42.13(38.03–46.23)	33.1(31.11–35.1)	92.51(91.57–93.44)	39.88(38.17–41.59)
cutoff = 7	49.75(46.7–52.8)	85.37(78.86–91.88)	35.12(31.05–39.19)	42.29(40.24–44.34)	88.74(87.82–89.66)	20.71(19.09–22.33)
Entire Sample						
cutoff = 5	75.45(72.59–78.31)	62.52(55.93–69.11)	37.97(33.68–42.26)	32.5(30.36–34.64)	91.42(90.67–92.16)	44.5(42.43–46.57)
cutoff = 6	72.87(69.99–75.75)	67.46(60.82–74.1)	40.33(36.26–44.4)	34.87(32.96–36.79)	91.23(90.31–92.15)	39.63(37.77–41.49)
cutoff = 7	44.96(41.87–48.05)	85.81(79.14–92.48)	30.77(26.37–35.17)	43.11(40.72–45.49)	86.7(85.79–87.62)	19.89(18.25–21.53)

Note: PPV = positive predictive value; NPV = negative predictive value;

*  = Proportion of the study sample with risk score above the cutoff value; The confidence intervals (CIs) for sensitivity, specificity, and predictive values were calculated using bootstrapping (1000).

### Model validation

Relationships of variables with CAN prevalence in the validation cohort were broadly similar to those in the exploratory set (Table 1). The CAN risk score, derived from the exploratory cohort, predicted CAN prevalence in the validation cohort and entire sample well, with an AUC of 0.777 (95% CI 0.733–0.806, Figure 1) and 0.740 (95% CI 0.712–0.768), respectively. In the validation cohort, at the cutoff point of 5, the sensitivity and specificity were 77.61 and 63.92% (Table 4), respectively. However, the optimum cutoff point for the validation set was 6 (sensitivity = 74.63%, specificity = 67.50% and % need subsequent testing = 39.63%). In entire sample, at the cutoff point of 6, the sensitivity and specificity were 72.87 and 67.46%, respectively. The specificity, predictive values, and the percentage of the population that needed subsequent testing were similar among the three sets, whereas the sensitivity tended to be higher in validation set compared with exploratory set. The distribution of the CAN risk score in entire sample sets was calculated. These was the highest proportion of individuals at score 5 (18.36% in the entire sample, data not shown). The CAN prevalence increased according to increasing score in entire sample. The highest CAN prevalence was more than 75.00% in subjects with score 15 (data not shown).

### Comparison of individuals with low- and high-risk scores

The cardiovascular and diabetic neuropathy risk profile was more unfavorable in the individuals with CAN in group with risk score (6 to 15) compared with those in group with low-risk score (0–5) (Table 5). The age and gender were similar the two groups (P>0.05 for all). Demographic parameters including BMI, SBP and DBP were significantly greater in individuals with CAN in group high-risk score as compared with those in group with low-risk score (P<0.001 for all). Blood glucose and insulin function parameters were significantly difference between the two groups (P<0.001 for all). The prevalence of HT, DM and MS were higher in individuals with high-risk score (P<0.001 for all). However, similar trends were seen for renal function profile.

**Table 5:** Comparison of individuals with CAN in low- and high-score groups.

Variables	Low score group (0–5)	High score group (6–15)	*P* value
N	105	282	-
Age	62.17±8.22	63.35±8.53	0.187
Gender	47 (44.76%)	115 (40.78%)	0.782
BMI	24.1±3.2	25.24±3.89	0.004
SBP	127.77±19.55	135.89±20	<0.001
DBP	78.32±9.15	82.92±10.11	<0.001
FPG	5.92±2.4	6.23±2.61	0.246
PBG	8.15±4.2	9.58±4.74	0.004
TC	5.27±1.00	5.45±1.07	0.118
TG	1.67±0.94	2.03±1.27	0.003
HDL	1.35±0.33	1.33±0.31	0.431
LDL	3.11±0.77	3.3±0.82	0.028
HR	69.91±6.63	85±9.58	<0.001
Smoking	16(15.24%)	46(16.31%)	0.604
HT	50(47.62%)	197 (69.86%)	<0.001
DM	29(27.62%)	113(40.07%)	0.004
MS	45(42.86%)	163(57.80%)	0.001

Note: BMI- Body mass index, SBP- systolic blood pressure, DBP- diastolic blood pressure, FPG- fasting plasma glucose, PBG- plasma blood glucose, TC- serum total cholesterol, TG- triglyceride, UA- uric acid, HDL- high-density lipoprotein cholesterol, LDL- low density lipoprotein cholesterol, HR-heart rate, MS- metabolic syndrome, HT- Hypertension, DM- Diabetes.

## Discussion

A large-scale population-based cross-sectional study was conducted to develop a practical tool for prediction of CAN among 2092 participators in Chinese population. The average age of total sample was more than 60 years old. Of a total of 2092 subjects, 46.65%, 21.33% and 39.82% subjects had HT, DM, and MS respectively. The prevalence of these three diseases and CAN (18.51%) in general population was similar with previous studies [17], [18], [19], [20]. The CAN prevalence was 31.17%, 24.69% and 24.49% 24.14% in subjects with the respective disease. This sample was a good represent one across the country, the model might work well in outside the studied areas in China. Development of CAN risk score system was conducted in exploratory set using univariate and multiple linear regression models. The final risk score system was validated in another set and entire sample. In final, a simple CAN risk score system was developed to screen CAN in general population.

The score system based on simple parameters included age, BMI, HT and resting HR. The simple model without laboratory tests is almost as good as models including HRV test. This risk score at cutoff of 6 of 15 can detect 72.87% of individuals with previously undiagnosed CAN. In addition, the risk score has a high specificity of 67.46% and decreases the proportion of individuals in the population that need subsequent testing to 39.73%. The risk score at cutoff of 5 has a higher sensitivity of 75.45%, but decreased specificity to 62.52% and increased the proportion of individuals in the population that need subsequent testing to 44.50%. ROC curves have been recommended to apply to find the optimal threshold in screening and diagnostic tests, due to the curves quantified as a single number through the AUC. Mathematically, the optimal threshold in ROC curves is defined as the point maximizing the sum of sensitivity and specificity. To our knowledge, large-scale study to explore the CAN risk score was little known. Lambrecht et al. developed a simple, rapid autonomic cardiovascular evaluation (RACE) to screen CAN in Caucasians [21]. In the study, 37 eligible patients undergoing full autonomic testing in the laboratory have been enrolled. A RACE score system was developed to screen CAN. The optimal score had a sensitivity of 85% but very low specificity of 30%. The proportion of the entire population needed subsequent testing was not analyzed due to small sample size. RACE does not satisfy the criteria to serve as a robust CAN screen. A computerized measurement system has been conducted for guided routine evaluation of CAN [22]. The portable computerized system was used for a guided step-by-step performance of several cardiovascular tests for autonomic neuropathy, which has been compared to the traditional method using an electrocardiograph (ECG). The new and traditional approaches were used among 74 patients with diabetes, respectively. The computerized measurement system had sensitivity of 70–90% and specificity of 71–90%. Similarly, the proportion of the entire population needed subsequent testing has not calculated due to study design. The approach can save amount of time and is also easy to use, and represents a valid alternative for the routine screening of autonomic neuropathy. However, the computerized measurement system was more complex, time-consumption and expensive as compared with CAN risk score system in our study.

This score system can be completed without any specific measurements, and therefore, the simple model is noninvasive, more convenient, and less expensive compared with the models that rely upon complex HRV tests or CA reflex tests. Additionally, the cost of each HRV was cheaper than other cardiovascular autonomic function tests in China. The risk score method is used for a primary medical care setting and for a layperson to perform self-assessment to identify high-risk people. The high-risk individuals were generally recommended to perform a HRV test that is available in most primary health care settings in China now. In addition, the high-risk individuals will benefit from receiving health education and changing to healthy lifestyles at an early stage so as to prevent or delay the onset of CAN. The present study provides a risk score based on a specific population in China. The predictive performance and discriminative ability of the score is not confirmed in other ethnics. Nevertheless, future researchers might investigate more about the generalizability of these score rules across countries.

Among the modifiable risk factors that played a substantial role in previous studies were resting HR, obesity measured by BMI, and BP [6], [18], [23]. Resting HR was the strongest predictor of CAN. Resting HR was considered as an outcome of CAN. If the HR of an individual was more than 90 beats/min, his risk score was at least up to 8. It means the person was a high-risk one, which was consisted with clinical early stage outcome of CAN [1], [6]. Controlling resting HR well might the most important for prevention of CAN complications. In this study, BMI was detected to increase CAN risk at cutoff points suggested for China populations that are lower than those used for people in Western countries [7], [24], [25]. BMI was strong positive correlated with IR and dyslipidemia, and was a strong independent predictor of CAN. In this score system, BMI was an indicator of IR and diabetes status that was the most contributors to CAN. High BP plays a crucial role in progression of CAN. Low HRV and CAN associated with HT. High-risk individuals might benefit from controlling BP to normal status. In general, DM and its duration were considered as two main risk factors for the progression of CAN. In this study, the two factors with high ORs associated with CAN. However, blood taking and undiagnosed DM were not suitable for a simple score system. Furthermore, CAN prevention researches should be performed in areas controlling these risk factors.

A risk score based on questions regarding phenotypical characteristics for CAN could never obtain a sensitivity of 100%. False-negative is mainly attributed to two factors that other risk factors apart from risk score system contribute to outcome, and the value of risk factor was not difference between false-negative and true-positive individuals. In this study, a part of individuals with CAN are not obesity, or have a normal resting HR due to both impaired sympathetic and the parasympathetic nervous system. The prevalence of CAN with normal resting HR in the population is little known. In addition, false-negative individuals were lower resting HR, indicating those people with long-term duration of CAN. In our risk score, 39% of individuals with previously undiagnosed diabetes will be missed. We found that false-negative individuals had lower PBG and LDL levels; however, there was no different in FPG and TC levels as compared with true-positive individuals. Furthermore, the resting HR and BP parameters were significantly difference between the two groups. This is partly because BMI, HT and HR score can not completely reflect these differences. In the risk score system, BMI was the strongest predictor of dyslipidemia; however, the risk factors can not completely represent contribution of lipid profile on CAN. Screening is an ongoing process, presumably, some of the false-negative individuals will become true positive over time and will be picked up in a subsequent screening.

Several limitations of the study deserve comment. First, the design of the present study was cross-sectional study and thus the temporal sequence between risk factors and outcome was questionable. Second, participants recruited from Shanghai and external validation has not been performed. So generalizability of our prediction model should be needed to determine. Finally, it is important to mention that our study was performed on Chinese individuals, and our findings may not be relevant to people of other ethnicities.

In conclusion, the simple CAN risk score developed here can be applied in a stepwise screening strategy. People with a high-risk score should be referred for further standard CA function tests and changes to a healthier lifestyle for primary prevention.
